# Polymeric antibubbles with strong ultrasound imaging capabilities†

**DOI:** 10.1039/d4cc03572k

**Published:** 2024-09-27

**Authors:** Roman A. Barmin, Jens Köhler, Michael Pohl, Bea Becker, Fabian Kiessling, Twan Lammers, Albert T. Poortinga, Roger M. Pallares

**Affiliations:** a Institute for Experimental Molecular Imaging, RWTH Aachen University Hospital Aachen 52074 Germany rmoltopallar@ukaachen.de tlammers@ukaachen.de; b DWI – Leibniz Institute for Interactive Materials Aachen 52074 Germany; c Polymer Technology, Eindhoven University of Technology Eindhoven 5612 AZ The Netherlands albert.poortinga@bether-encapsulates.nl

## Abstract

Antibubbles are liquid droplets encapsulated by a gas film that have recently been explored for on-demand ultrasound-triggered drug release. However, their ultrasound imaging capabilities are limited by their stiff shells stabilized with silica nanoparticles. Here, we develop polymeric antibubbles that generate greater ultrasound contrast than silica-based antibubbles, while showing better stability than conventional polymeric microbubbles.

## Abstract 2

Antibubbles are liquid droplets encapsulated by a gas film and are proposed for ultrasound-triggered drug release. Here, we develop polymeric microbubbles with greater ultrasound imaging response than conventional Pickering-stabilized antibubbles.

## Graphical abstract

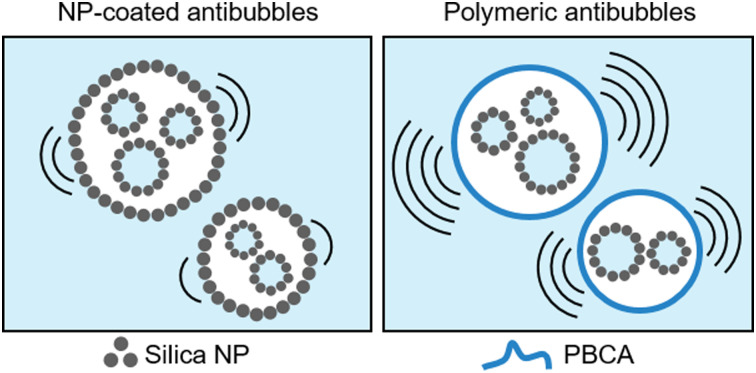

Ultrasound (US) is used for many different purposes, ranging from synthetic chemistry to clinical imaging and therapy, therefore, US-responsive materials are of growing interest because of their ability to interact with acoustic waves and facilitate or enhance processes mediated by US.^1^ Recently developed antibubbles (so-called “inverted microbubbles”) consist of liquid droplets wrapped in a film of air in a liquid, resulting in a water-in-air-in-water structure.^2^ These colloidal particles combine the US responsiveness and ability to maintain stable cavitation under US pulses provided by the entrapped gas phase with the large loading capacity of the inner core droplets, which can occupy up to 21% of the total antibubble volume.^3^ Consequently, antibubbles have been proposed as delivery systems for on-demand US-triggered drug release, and recent reports have shown that antibubbles enable tunable payload release under therapeutic low intensity US.^4^ However, current antibubble formulations are limited by their poor US imaging features. This can be attributed to the fact that the inner and outer shells of the antibubbles are mainly stabilized with solid silica nanoparticles (SiNP) through so-called Pickering stabilization, which tend to form stiff and rigid films with limited oscillation capabilities.^3^

In contrast, gas-filled microbubbles (MB) of 1–8 μm in diameter display excellent acoustic responses, and are extensively used in the clinic as contrast agents for US imaging at frequencies of 1–18 MHz and peak negative pressures below 3 MPa.^1^ MB are also being explored in clinical trials for molecular imaging and therapeutic sonopermeation.^5^ Although most MB applications are related to pathologies in the vasculature, there is increasing clinical interest in using MB (and other US contrast agents) for the diagnosis of non-vascular disorders, such as the assessment of vesicoureteral reflux^6^ or tubal patency.^7^ Furthermore, MB and other acoustic responsive materials have been explored for US-mediated bacterial film disruption at the preclinical level, owing to their great oscillation capabilities.^5^ Among the different shell materials available, poly(butyl cyanoacrylate) (PBCA) is particularly advantageous, since it is biocompatible and already FDA-approved for surgical glue applications.^8,9^ Hence, US-responsive PBCA MB are currently being translated into the clinic owing to their adequate acoustic responses and functionalization capabilities.^10^ In addition, the acoustic response of the PBCA MB platform can be tailored by manipulating their polymer chemistry.^11^ Therefore, we hypothesize that the introduction of PBCA chains to stabilize the antibubble structure may serve as a strategy to improve US imaging capabilities of antibubbles.

In this study, we demonstrate that the outer shell of antibubbles can be formulated using a polymeric shell of entangled PBCA chains instead of a Pickering emulsion made of SiNP. The resulting PBCA-coated antibubbles showed significantly greater US contrast enhancement compared to SiNP-coated counterparts. While PBCA MB tended to burst immediately upon application of high acoustic power, both PBCA and SiNP-coated antibubble formulations remained intact and demonstrated stable contrast signal generation. These findings provide the basis for the design of new polymer-coated antibubbles with improved performance in US imaging and therapy where strong acoustic responses are required.

All antibubble formulations were synthesized using the multi-step process shown schematically in Fig. S1 and described in detail in the ESI,† while different antibubble variants are shown schematically in Fig. 1. PBCA-coated antibubble samples were prepared at the homogenization rate of 5000 rpm during double emulsion formation and labeled to indicate the pH of the W_2_ aqueous phase (PBCA 2.5 and PBCA 7.0), while control SiNP-coated samples were labeled to indicate the homogenization rate of 5000 rpm and 10 000 rpm used to form the double emulsion (SiNP 5k and SiNP 10k).

**Fig. 1:**
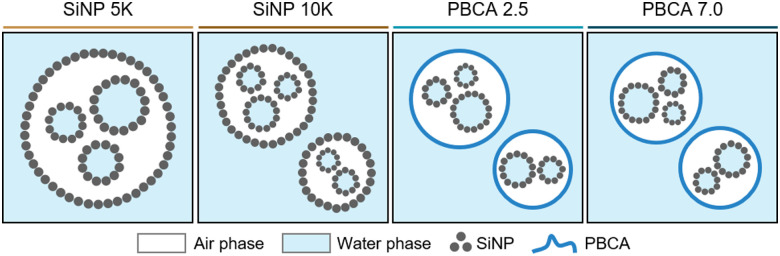
Schematic representation of the antibubble variants. A flowchart of the antibubble production and a schematic representation of the resulting antibubble samples are shown in Fig. S1 (ESI†).

Once the antibubble samples were prepared, optical microscopy (OM), confocal laser scanning microscopy (CLSM) and scanning electron microscopy (SEM) were employed to characterize the resulting microparticles, and representative images are shown in Fig. 2a. The OM images confirmed the spherical shape of the antibubbles, while the dark cores within the particles suggested the presence of a gaseous phase within each particle similarly to previous reports on antibubbles.^4^ Furthermore, the multi-component interior of the antibubbles was observed in the CLSM images, as the fluorescent dye calcein was introduced to the first aqueous phase during their synthesis. The presence of stabilizing SiNP within the fragmented antibubbles was also visible in the SEM images. In the case of the antibubbles SiNP 5k and 10k, both formulations showed smooth shell surfaces, which were consistent with previous reports on Pickering stabilized antibubbles.^3^ Taken together, these observations confirmed the antibubble colloidal structure of both PBCA-coated and SiNP-stabilized samples. Based on the microscopy results, we could not identify any colloidal microparticles without SiNP in their cores.

**Fig. 2:**
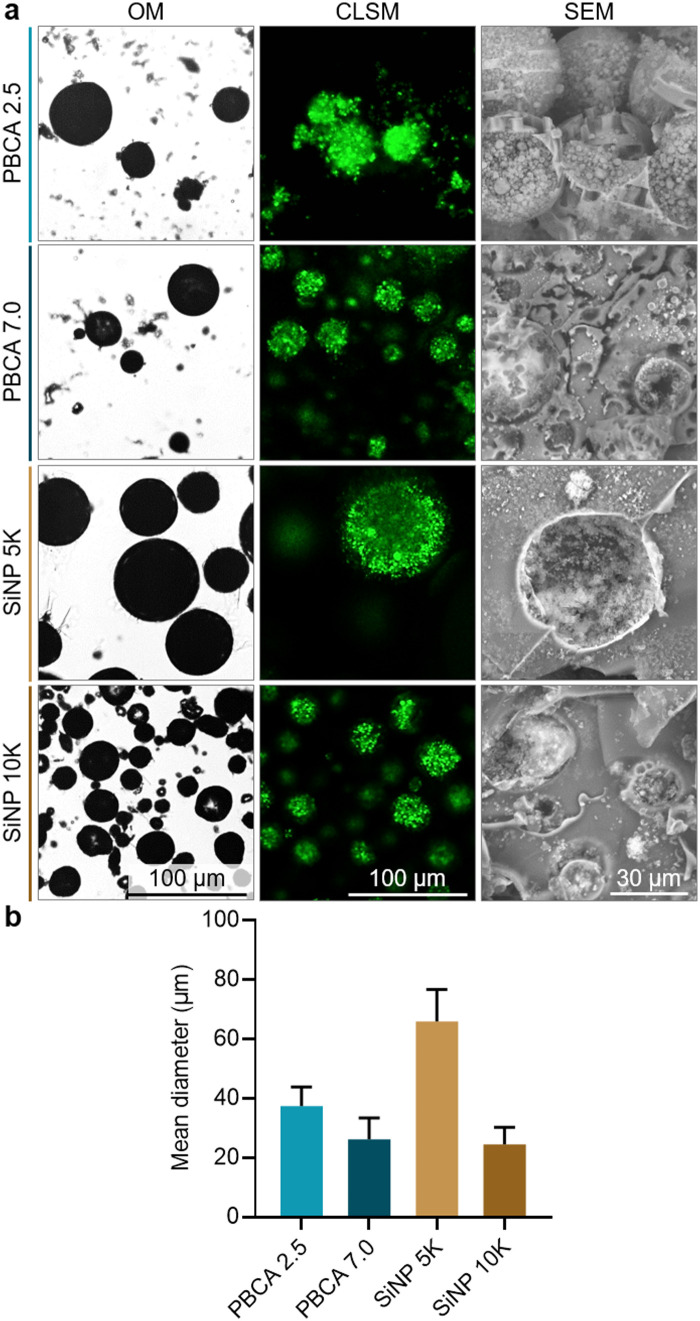
Characterization of the different antibubbles. (a) Representative optical microscopy (OM), confocal laser scanning microscopy (CLSM), scanning electron microscopy (SEM) micrographs, and (b) mean diameter of antibubble samples. Wide-area CLSM micrographs of antibubbles are shown in Fig. S2 (ESI†).

The polymeric antibubbles displayed smaller mean diameters in comparison to their SiNP-coated counterparts (shown in Fig. 2b). While the parent double emulsions of PBCA-coated antibubbles and SiNP 5k were all homogenized at 5000 rpm, the SiNP 5k antibubbles showed a mean diameter of 66 ± 10 μm, while the PBCA 2.5 and PBCA 7.0 mean diameters were 37 ± 6 μm and 26 ± 7 μm, respectively. This could be due to the lower interfacial tension of the PVA-containing solutions compared to the SiNP-based solutions used during antibubble synthesis (Table S1, ESI†). In addition, the antibubbles PBCA 7.0 might have had a smaller mean diameter compared to the antibubbles PBCA 2.5 due to the faster BCA polymerization at higher pH, which gave the emulsions less time to coarsen due to the coalescence of oil droplets with each other or inner water droplets with the surrounding water phase.^12^ The mean diameter of the PBCA-based antibubbles was comparable to that of SiNP 10k sample (24 ± 6 μm), where the parent double emulsion was homogenized at the higher speed of 10 000 rpm during the synthesis.

Proton nuclear magnetic resonance (^1^H NMR) spectroscopy, gel permeation chromatography (GPC), and Fourier transform infrared (FTIR) spectroscopy were performed to further investigate the composition of the polymeric antibubbles. For NMR and GPC analyses, antibubble and PBCA MB powders were dissolved in chloroform, and the undissolved fraction was removed by filtration prior the measurements. The ^1^H NMR spectra of the samples demonstrated that the shell of the polymeric antibubbles (as well as the PBCA MB used as a reference) consisted of PBCA chains, as shown in Fig. 3a. In addition, BCA polymerization could be confirmed by the absence of peaks originating from the monomeric BCA vinyl group (at 6.57 and 6.99 ppm), as described in the literature.^13^

**Fig. 3:**
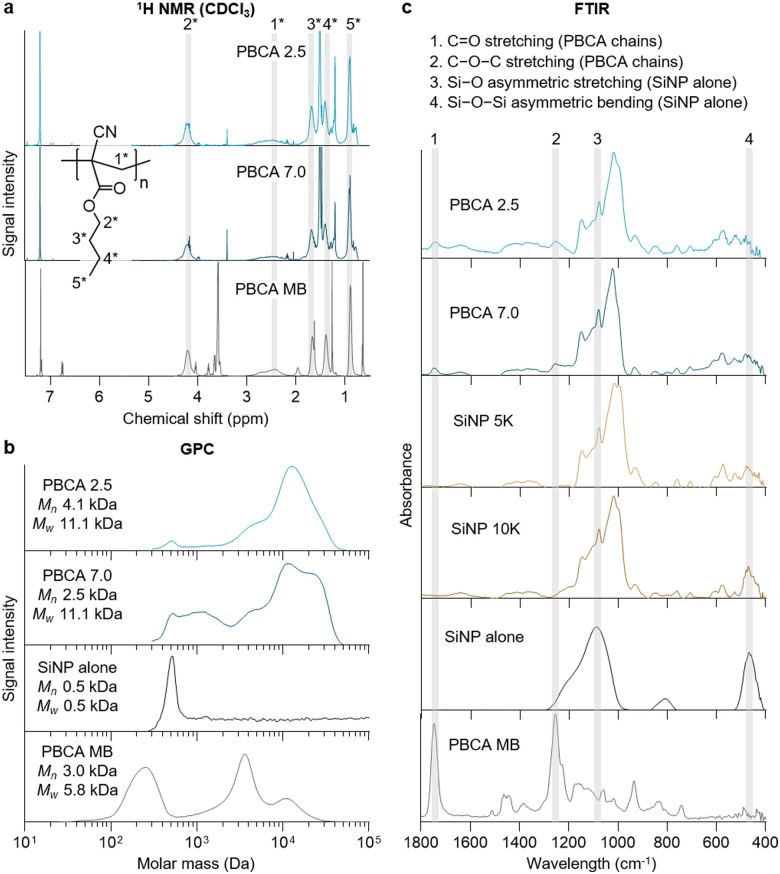
Analysis of the antibubble chemical composition. (a) Proton nuclear magnetic resonance (^1^H NMR) spectra with peak assignment, (b) molar mass distribution profile provided by gel permeation chromatography (GPC), and (c) Fourier transform infrared (FTIR) spectra of polymeric antibubbles and MB confirm the presence of PBCA chains in the samples, as well as the presence of SiNP in all synthesized antibubble formulations.

GPC molar mass distribution profiles of polymeric antibubbles showed that the shells were composed of polymer chains with weight average molar mass (*M*_w_) values below 40 kDa (Fig. 3b), which is the size cutoff for renal clearance.^11^ The signal around 500 Da confirmed the presence of SiNP in the polymeric antibubble samples, while this band was absent in PBCA MB (as the MB reference did not contain SiNP). The GPC chromatogram for PBCA 2.5 showed a bimodal molecular weight distribution (*M*_p_ = 4 kDa and 11 kDa). In comparison, PBCA 7.0 displayed a broad multimodal molecular weight distribution. Similar main signals at *M*_p_ of 4 kDa and 11 kDa were present, but significant fractions with molecular weights around 1.2 kDa and 23 kDa, respectively, could also be observed. This agrees with studies that report more controlled and slow polymerization of BCA under acidic conditions compared to rapid polymerization occurred under basic conditions.^13^ Both polymeric antibubbles had larger molar masses of PBCA chains compared to the PBCA MB sample, which could be due to the replacement of Triton X-100 (small molecule surfactant used in PBCA MB synthesis) with macromolecular PVA chains of 30–70 kDa for the antibubble synthesis.^8^

Antibubble and MB powders were analyzed without reconstitution by FTIR (Fig. 3c). In both PBCA- and SiNP-stabilized antibubble samples, bands associated with SiNP, namely 1092–1086 cm^−1^, representing Si–O asymmetric stretching, and 474–456 cm^−1^, representing Si–O–Si asymmetric bending, were observed.^14^ In addition, representative bands of the ester unit within the PBCA chain structure in both polymeric antibubbles and PBCA MB were present. The bands were associated with oxygen-containing groups within the backbone of the polymer, namely C=O stretching (∼1740 cm^−1^) and C–O–C stretching (∼1252 cm^−1^).^15^ Taken together, these results corroborated the presence of PBCA chains and silica within the polymeric antibubble samples.

We further investigated the US imaging capabilities of the produced antibubbles using the Vevo3100 preclinical system, which is equipped with a transducer with a central frequency of 18 MHz. We embedded the antibubbles in gelatin phantoms and used the PBCA MB with a mean diameter of 2.3 ± 0.5 μm as a reference sample with known acoustic properties.^11^Fig. 4a shows the sonographic images of the different samples in the US non-linear contrast (NLC) mode, which is specific to the acoustic response of gas-filled agents under US pulses. At 4% acoustic power as provided by the setup manufacturer, both polymeric antibubbles exhibited greater acoustic response compared to their SiNP-stabilized counterparts, as shown in Fig. 4b. The low contrast signal provided by SiNP-coated antibubbles was consistent with a previous report.^3^ However, all antibubble samples had lower NLC intensities compared to PBCA MB. Although PBCA MB had a smaller mean diameter compared to antibubble samples, their cores were entirely filled with air. In contrast, the antibubble cores entrapped particles, which might have limited the vibration capabilities of the antibubble system compared to the MB. The absence of signal loss at 100% acoustic power suggested that all antibubble formulations remained stable under US pulses, as shown in Fig. 4c. This is consistent with the previously described stable cavitation behavior of antibubbles under mechanical index values up to 1.2.^3^ When 100% acoustic power was applied, the polymeric antibubbles retained their greater acoustic response compared to the SiNP-coated counterparts. Moreover, all antibubble samples provided a better NLC signal than the PBCA MB (Fig. 4d). This observation was likely caused by the gradual destruction of the PBCA MB under high acoustic power^10^ and was further validated by measuring the NLC signal of the samples under continuous US pulse irradiation (Fig. 4e). In contrast, all antibubble formulations retained their ability to vibrate under US pulses at high power. The lack of signal loss suggested that the antibubbles remained stable at both low and high acoustic power levels, whereas PBCA tended to burst gradually at high acoustic power. Therefore, these results demonstrated that replacing the outer silica shell of the antibubbles with PBCA chains enhanced the US contrast of the formulations, while preserving the antibubble greater acoustic stability compared to the standard PBCA MB.

**Fig. 4:**
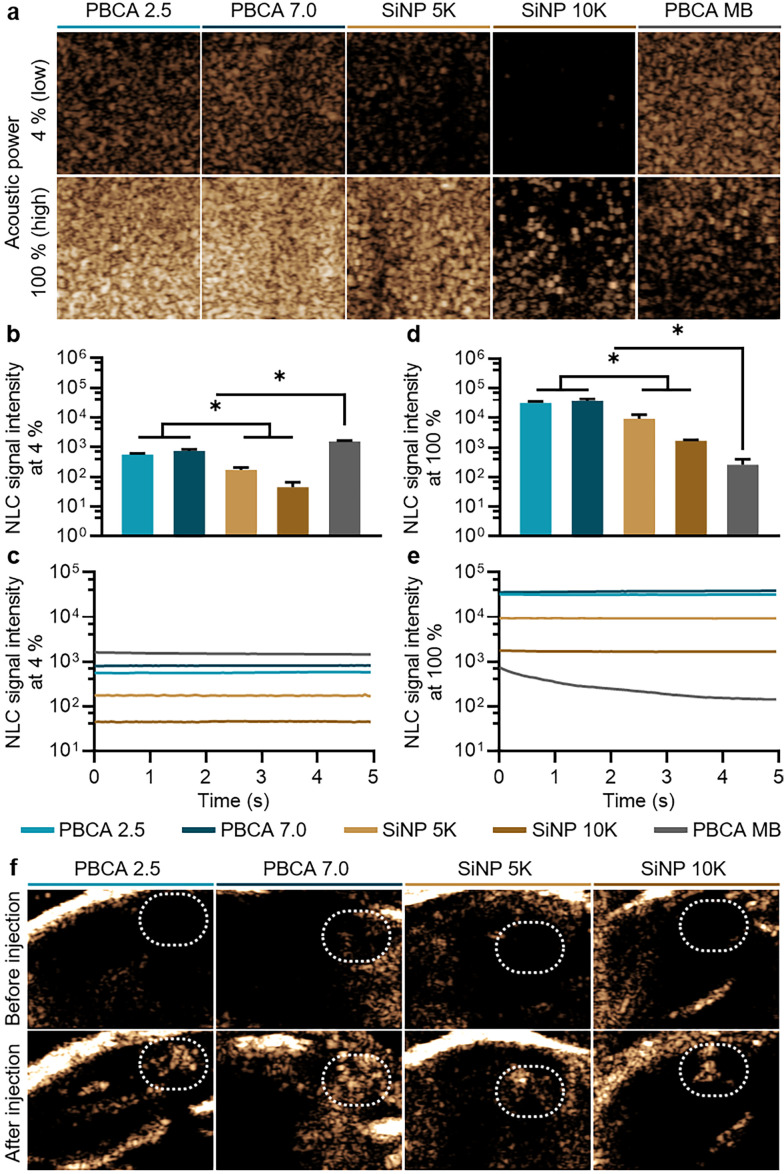
US performance of antibubbles and PBCA MB. (a) Representative US NLC mode images of samples at 4% and 100% acoustic power obtained in phantoms. (b) Mean NLC signal intensities and (c) NLC signal intensity over time at 4% acoustic power. (d) Mean NLC signal intensities and (e) NLC signal intensity over time at 100% acoustic power. (f) Representative *ex vivo* US NLC mode images at 100% acoustic power. Regions of interest are indicated by the white dashed line. (*) indicates groups that are significantly different with *p* < 0.05 between compared groups (one-way ANOVA with *post hoc* Tukey HSD test), respectively.

For *ex vivo* proof-of-concept US imaging, legs of deceased mice were intramuscularly injected with the antibubble samples (representative US NLC images are shown in Fig. 4f). The contrast enhancement levels were consistent with the ones obtained in gelatin phantoms, and all antibubbles maintained stable NLC intensity over 100 s (Fig. S3, ESI†), confirming the acoustic stability of the antibubbles and enhanced US imaging capabilities of the PBCA-coated formulations.

In summary, we stabilized antibubbles with polymeric shells made of PBCA chains instead of SiNP particles. The introduction of the polymer outer shell improved the acoustic response of the antibubbles compared to the SiNP-based counterparts. Preservation of the acoustic signal under high acoustic power without destruction of the antibubbles paves the way for potential applications, such as non-vascular disorder diagnosis or US-mediated bacterial film disruption, where strong acoustic performance and high stability (under high acoustic power) are required. Further developments in reducing the mean diameter of polymeric antibubbles, as well as better polymerization control may unlock the full potential of polymeric antibubbles for US imaging.

This work was funded by the Federal Ministry of Education and Research (BMBF) and the Ministry of Culture and Science of the German State of North Rhine-Westphalia (MKW) under the Excellence Strategy of the Federal Government and the Länder, by the European Research Council (ERC CoG 864121, Meta-Targeting), the European Commission (EuroNanoMed-III: NSC4DIPG), and the German Research Foundation (DFG: GRK 2375 (grant #331065168) and SFB 1066).

## Data availability

The supporting data has been included in the ESI.†

## Conflicts of interest

F. Kiessling, and T. Lammers are among the co-founders of the SonoMAC GmbH that produces polymeric microbubbles. F. Kiessling is a consultant of Fujifilm Visualsonics. A. T. Poortinga is the founder of Bether Encapsulates B.V., which aims to commercialize antibubbles.

## Supplementary Material

CC-060-D4CC03572K-s001
