# Supplementary material for: Polymeric antibubbles with strong ultrasound imaging capabilities
Source: Chem Commun (Camb). 2024 Sep 27;60(91):13340–3. doi: 10.1039/d4cc03572k (PMC11427993; doi:10.1039/d4cc03572k)
Supplement: CC-060-D4CC03572K-s001 [file CC-060-D4CC03572K-s001.pdf]

Supplementary Information (SI) for ChemComm.
This journal is © The Royal Society of Chemistry 2024

- SUPPORTING INFORMATION –

Polymeric antibubbles with strong ultrasound imaging capabilities

Roman A. Barmin,[a] Jens Köhler,[b] Michael Pohl,[b] Bea Becker,[b]
Fabian Kiessling,[a] Twan Lammers,[a,*] Albert Poortinga,[c,*] Roger M. Pallares[a,*]

[a] Institute for Experimental Molecular Imaging, RWTH Aachen University Hospital, Aachen 52074, Germany; Email: rmoltopallar@ukaachen.de; tlammers@ukaachen.de

[b] DWI – Leibniz Institute for Interactive Materials, Aachen 52074, Germany

[c] Polymer Technology, Eindhoven University of Technology, Eindhoven 5612 AZ, the Netherlands; Email: albert.poortinga@bether-encapsulates.nl

Table of Contents

Experimental section S2

Figure S1. Flow chart of antibubble production and schematic representation of the resulting antibubble samples S6

Figure S2. Representative wide-area CLSM micrographs of different antibubble variants S8

Table S1. Properties of solutions with stabilizers used for antibubble preparation S9

Figure S3. Representative *ex vivo* NLC signal intensities of the antibubble samples over time at 100 % acoustic power S10

References S11

EXPERIMENTAL SECTION

Materials

Butyl cyanoacrylate (BCA) was purchased from Special Polymer Ltd. (Sofia, Bulgaria). The hydrophobized fumed silica particles, HDK H17 and AEROSIL R816, were provided by Wacker (Munich, Germany) and Evonik (Essen, Germany), respectively. Calcein, sodium chloride (NaCl), cyclooctane, poly(vinyl alcohol) (PVA) with average molecular weight of 30 – 70 kDa and hydrolysis degree of 87 – 90 %, Triton X-100, and gelatin were purchased from Sigma-Aldrich (Munich, Germany). 2DE maltodextrin (MD) was purchased from Barentz BV (Hoofddorp, the Netherlands). Deionized (DI) water was produced by a PURELAB flex 2 device from ELGA LabWater (Celle, Germany) and used for all experiments. All reagents were of appropriate analytical grade.

Antibubble and microbubble synthesis

Antibubble synthesis was performed similar to previous reports[1–3] with modifications required for BCA polymerization. The formation of double emulsion ($W_1/O/W_2$) was carried out through a two-step homogenization process. A primary emulsion (W_1/O) was obtained by dispersing 6 g of DI water (containing 10 % (w/v) MD, 0.5 % (w/v) NaCl and 1 % (w/v) calcein as a fluorophore) in 24 g of cyclooctane oil containing 5 % (w/v) SiNP (H17 hydrophobized fumed silica particles) and homogenizing with an Hielscher UP100 ultrasonic sonotrode (Hielscher Ultrasonics GmbH, Teltow, Germany) for 30 s until no visible SiNP were observed. Cyclooctane was used as oil phase to facilitate its replacement with air from the parent double emulsions during the freeze-drying step,[4] thus allowing the formation of antibubbles. Both W_1 and W_2 aqueous phases were prepared with 10 % maltodextrin (MD) and 0.5 % NaCl to provide the glass-forming properties required for lyophilization and to equilibrate the internal and external osmotic pressures, respectively. For polymeric-based samples, 1% (w/v) BCA monomer was subsequently added to the oil phase after the primary emulsion was produced to promote the formation of PBCA antibubbles.

Next, three external water phases (W_2) were prepared containing 10 % MD (w/v), 0.5 % (w/v) NaCl and two different additives: 0.5 % (w/v) SiNP (R816 hydrophobized fumed silica particles) for Pickering emulsion-based samples or 0.5 % (w/v) PVA with the pH adjusted to 2.5 or 7.0 for PBCA-coated samples. Such synthesis conditions were chosen based on a previous protocol that reported the miniemulsion polymerization of BCA monomers in the presence of surfactant-coated templates.[5] PVA was chosen as the surfactant required to direct the BCA polymerization as reported in previous studies.[6,7] Two different pH levels were explored during the antibubble synthesis, since pH controls the BCA polymerization rate.[8] At

pH 7.0, the polymerization rate is rapid (within a few min), while at pH 2.5, BCA tends to polymerize slowly, within tens of min.[9,10] Once all the W_2 phases were ready, the double ($W_1/O/W_2$) emulsions were prepared by homogenizing 2 g of previously prepared primary emulsion in 20 g of corresponding SiNP or polymer containing W_2 phase. The secondary emulsification step was carried out using an Ultra-Turrax T25 (IKA-Werke, Staufen, Germany) for 30 s. SiNP-based samples were emulsified at 5,000 and 10,000 RPM that are commonly used during the formation of the double emulsions,[1,2] since they render antibubbles with different mean diameters. PBCA-based samples were homogenized at 5,000 RPM. For PBCA-based samples, an additional step was performed: 1 % (w/v) BCA was added dropwise into the W_2 phase after the homogenization and the solutions were mixed for 30 min to allow monomer polymerization at the O/W_2 interface of prepared $W_1/O/W_2$ emulsions.

The produced double emulsions were quickly frozen in −80 °C ultra-freezer (BINDER GmbH, Germany). Afterwards, the frozen emulsion samples were lyophilized at −85 °C (0.01 mbar vacuum) using a freeze dryer (LyoAlfa 15, Telstar, Germany) for 48 h. Subsequently, the antibubbles (i.e., $W_1/A/W_2$) were produced by rehydration of the freeze-dried material with an aqueous solution containing 10 % (w/v) MD and 0.5 % (w/v) NaCl. PBCA-coated antibubbles are labeled according to the pH of the W_2 phase (2.5 or 7.0) as PBCA 2.5 and PBCA 7.0, respectively. Correspondingly, SiNP-based antibubbles are labeled according to the homogenization speed as SiNP 5K and SiNP 10K, respectively.

PBCA MB were synthesized via anionic polymerization of BCA monomers in the presence of hydroxyl ions as previously described.[11,12] Briefly, an aqueous solution of 1 % (v/v) Triton X-100 was prepared, and the pH of the solution was adjusted to 2.5. Next, 1 % (v/v) of BCA were added dropwise to the solution at room temperature, and the resulting mixture was stirred for 1 h at 10,000 RPM using an Ultra-Turrax T50 (IKA-Werke, Staufen, Germany). The produced suspension of air-filled PBCA MB was purified by three centrifugation steps at 500 RPM for 20 min and stored in a 0.02 % (v/v) Triton X-100 aqueous solution at pH 7.

Antibubble morphology characterization

Wide-field optical microscopy (OM) using a Zeiss Axiovert 40 C with an LD A-Plan 40x/0.5 Ph2 objective (Carl Zeiss Microscopy GmbH, Jena, Germany) and a blood counting chamber (ISOLAB Laborgeräte GmbH, Eschau, Germany) were used to estimate the concentration and mean diameter distribution of each sample. At least 50 separate particles were evaluated to quantify the mean diameter of each sample with ImageJ software (National Institutes of Health, USA).

Fluorescence microscopy was performed to assess the inner composition of the antibubble variants. The images were taken using a Leica TCS SP8 microscope (Leica Microsystems GmbH, Wetzlar, Germany).
The surface morphology and the interior of the antibubble variants was examined using a scanning electron microscope (SEM) setup Hitachi TM3030Plus (Hitachi, Ltd, Tokyo, Japan). The lyophilized $W_1/O/W_2$ emulsions were removed from the vial and crushed into small pieces using a surgical knife. The samples were glued to sticky carbon tape to the substrate, examined in the SEM at various magnifications and the images were recorded.

Interfacial tension measurements

Solutions containing the stabilizers used for antibubble synthesis (namely PVA chains and SiNP dispersed in DI water in the presence or absence of MD) were prepared, and the interfacial tension values were determined by a DSA100 Krüss GmbH (Hamburg, Germany) using the pendant drop type and Young-Laplace pressure methods. All measurements were carried out at room temperature.

Antibubble composition analysis

Proton nuclear magnetic resonance spectroscopy (^{1}H NMR), gel permeation chromatography (GPC) and Fourier transform infrared (FTIR) spectroscopy were performed to confirm the presence of PBCA chains and silica in the polymeric antibubbles. Antibubble powders were collected without reconstitution in an aqueous solution of 10 % MD, while PBCA MB (taken as a reference) were lyophilized using an Alpha 2-4 LD PLUS (Martin Chirist, Germany) for 48 h. Each powder sample was dissolved in deuterated chloroform ($CDCl_3$) or high-performance liquid chromatographic grade chloroform stabilized with 2-methyl-2-buthene (VWR, USA) for ^{1}H NMR and GPC measurements, respectively, and the undissolved portion was removed prior to measurements. For FTIR analysis, antibubble and MB powders were collected without reconstitution.
^{1}H NMR spectra were recorded on a Bruker Avance III 400 MHz spectrometer (Bruker, Germany).
A GPC set up, which included PU-2080 plus high-performance pump (Jasco, Pfungstadt, Germany), RI-2031 plus refractive index detector (Jasco, Pfungstadt, Germany), Sedex 85 evaporative light scattering detector (Sedere, Alfortville, France), one pre-column of 8 × 50 mm^2 and four SD Plus gel columns of 8 × 300 mm^2 (MZ Analysentechnik, Mainz, Germany), were utilized. Measurements were performed at 40 °C and 1 mL/min flow rate with gel particles

of 5 µm and pore width of 50, 100, 1000 and 10,000 Å. The calibration was done with polystyrene beads (Polymer Standards Service, Waldbronn, Germany).
FTIR spectra were measured using a Spectrum 3 FTIR spectrometer (PerkinElmer, Waltham, USA) in the 4,000 – 400 cm^{-1} range.

In vitro US imaging of antibubbles and MB in phantoms

US imaging of the produced formulations was performed using custom-made gelatin phantoms. Briefly, 5×10^5 particles (antibubbles or MB, respectively) were gently dispersed in 4.5 mL of 2 % (w/v) gelatin, which was embedded in 10 % (w/v) gelatin bulk and kept overnight at 4 °C to solidify. A preclinical Vevo 3100 US device (VisualSonics, Amsterdam, The Netherlands) was employed to image the samples in gelatin phantoms using the non-linear contrast (NLC) mode at a center frequency of 18 MHz, with 4 % (low) and 100 % (high) power that correspond to mechanical index values of 0.03 and 0.7, respectively. The NLC signals were recorded for 5 s for each sample. A region-of-interest of approximately 25 mm^2 was drawn at the middle of all images, and the resulting NLC intensity was analyzed using the Vevo LAB software.

Ex vivo US imaging

Ex vivo US imaging of mouse legs was performed using the Vevo 3100 US device, the MX 250 transducer with a center frequency of 18 MHz, and 100 % acoustic power. The C57BL/6J female mouse cadavers were reused from a previous experiment; therefore, no live animals were required or euthanized for the current study. Prior to intramuscular antibubble administration, control images were acquired for 100 s. After recording the control images, 1×10^6 particles of antibubble samples were injected in each leg, and US were recorded at nearly similar transducer positions as for the control images and similar setup settings as for the controls. For each sample, 3 mouse legs were imaged before and after antibubble injection. A region-of-interest of approximately 6 mm^2 was drawn for each image, and the resulting NLC intensity was analyzed using the Vevo LAB software.

Statistical analysis

Each formulation was measured three times. All values are presented as mean ± standard deviation. Statistical analyses were performed using GraphPad Prism 8. Figure 4 was analyzed using one-way ANOVA with post hoc Tukey HSD test. A p value of less than 0.05 was considered to be statistically significant, and (*) indicates groups that are significantly different with $p < 0.05$.

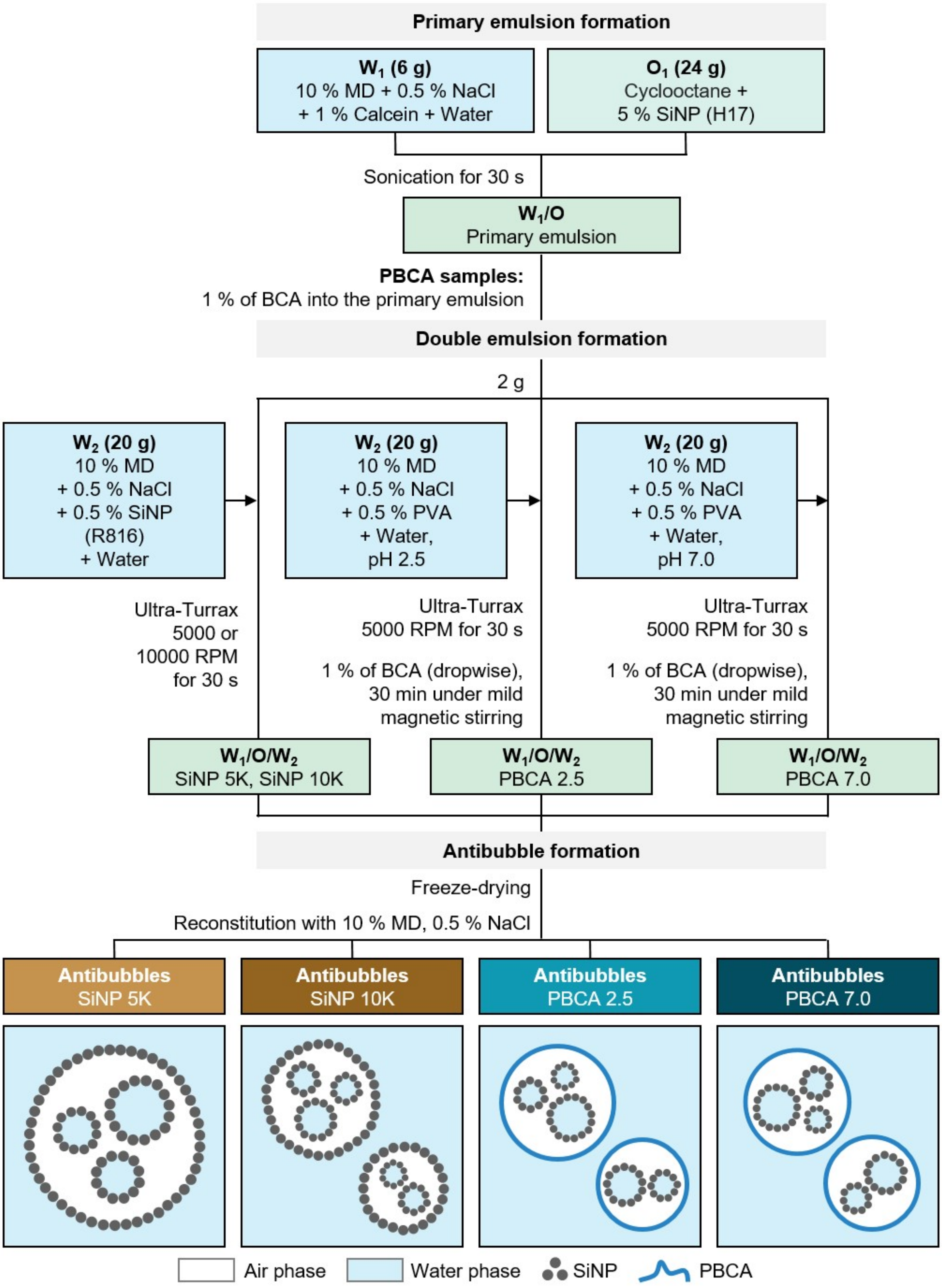

Figure S1. Flow chart of antibubble production and schematic representation of the resulting antibubble samples. The primary emulsion was formed similarly to previous reports, while for PBCA-based samples, a butyl cyanoacrylate (BCA) monomer (1 %) was subsequently added to the oil (O) phase. For double emulsion formation, the aqueous (W) phase containing

0.5 % poly(vinyl alcohol) (PVA) was adjusted to pH 2.5 or 7.0, then a BCA monomer (1 %) was added dropwise to the water phase and allowed to polymerize within 30 min under mild magnetic stirring. After lyophilization and reconstitution, two PBCA-coated antibubble formulations were obtained depending on the pH of the outer aqueous phase (PBCA 2.5 and PBCA 7.0), while Pickering emulsion based antibubbles prepared at different Ultra-Turrax speeds were used as references (SiNP 5K and SiNP 10K). All antibubble samples were produced and reconstituted with aqueous phases containing 10 % maltodextrin (MD) and 0.5 % NaCl. Percentages of all compounds are given as (w/v).

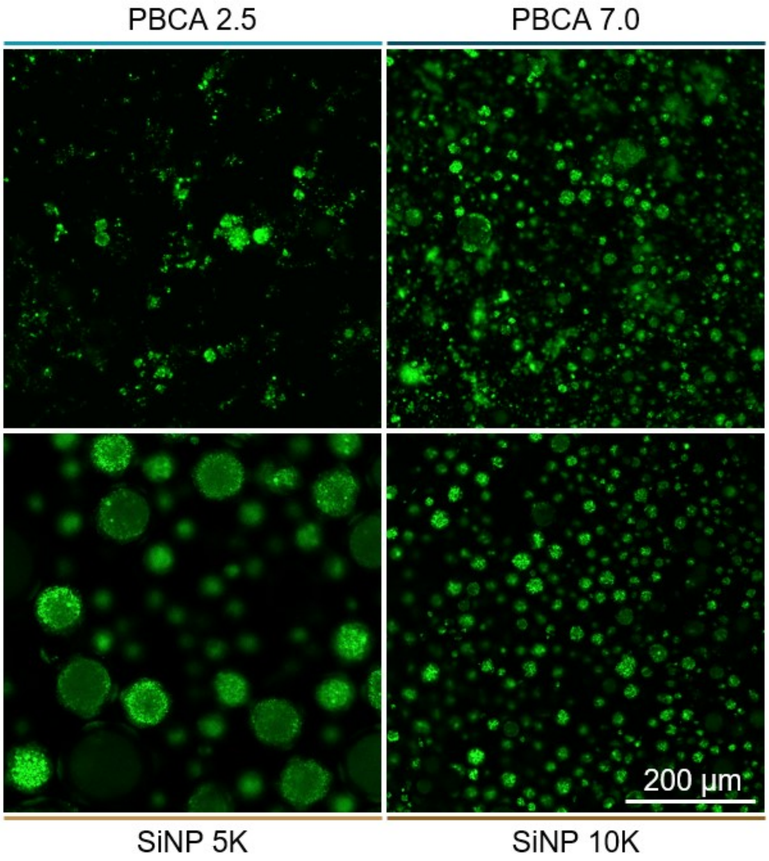

Figure S2. Representative wide-area CLSM micrographs of different antibubble variants.

Table S1. Properties of solutions with stabilizers used for antibubble preparation. Values represent mean ± standard deviation of each solution, measured in triplicates.

**Samples**	**Interfacial tension (mN/m)**
0.5 % NaCl	76.4 ± 0.5
0.5 % NaCl + 0.5 % PVA	54.3 ± 2.1
0.5 % NaCl + 0.5 % SiNP	77.0 ± 4.2
10 % MD + 0.5 % NaCl	70.0 ± 2.3
10 % MD + 0.5 % NaCl + 0.5 % PVA	50.2 ± 0.5
10 % MD + 0.5 % NaCl + 0.5 % SiNP	62.8 ± 2.2

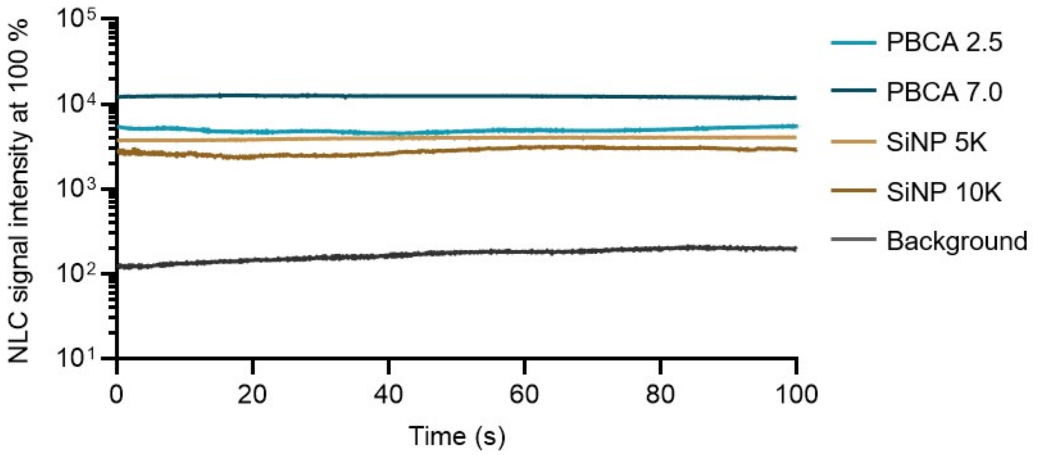

Figure S3. Representative *ex vivo* NLC signal intensities of the antibubble samples over time at 100 % acoustic power.

REFERENCES

1 S. Kotopoulis, C. Lam, R. Haugse, S. Snipstad, E. Murvold, T. Jouleh, S. Berg, R. Hansen, M. Popa, E. Mc Cormack, O. H. Gilja and A. Poortinga, *Ultrason. Sonochem.*, 2022, **85**, 105986.

2 R. Zia, A. T. Poortinga, A. Nazir, M. Ayyash and C. F. van Nostrum, *J. Colloid Interface Sci.*, 2023, **652**, 2054–2065.

3 J. Jiang, A. T. Poortinga, Y. Liao, T. Kamperman, C. H. Venner and C. W. Visser, *Adv. Mater.*, 2023, **35**, 2208894.

4 N. Moreno-Gomez, A. G. Athanassiadis, A. T. Poortinga and P. Fischer, *Adv. Mater.*, 2023, **35**, 2305296.

5 C. Limouzin, A. Caviggia, F. Ganachaud and P. Hémery, *Macromolecules*, 2003, **36**, 667–674.

6 A. Mitra and S. Lin, *J. Pharm. Pharmacol.*, 2003, **55**, 895–902.

7 J. Nicolas and P. Couvreur, *WIREs Nanomedicine and Nanobiotechnology*, 2009, **1**, 111–127.

8 B. H. Wang, M. Boulton, D. H. Lee, D. M. Pelz and S. P. Lownie, *J. Neurointerv. Surg.*, 2018, **10**, 150–155.

9 N. Behan and C. Birkinshaw, *Macromol. Rapid Commun.*, 2000, **21**, 884–886.

10 F. Hansali, G. Poisson, M. Wu, D. Bendedouch and E. Marie, *Colloids Surfaces B Biointerfaces*, 2011, **88**, 332–338.

11 R. A. Barmin, A. Dasgupta, C. Bastard, L. De Laporte, S. Rütten, M. Weiler, F. Kiessling, T. Lammers and R. M. Pallares, *Mol. Pharm.*, 2022, **19**, 3256–3266.

12 R. A. Barmin, A. Dasgupta, A. Rix, M. Weiler, L. Appold, S. Rütten, F. Padilla, A. J. C. Kuehne, A. Pich, L. De Laporte, F. Kiessling, R. M. Pallares and T. Lammers, *ACS Biomater. Sci. Eng.*, 2024, **10**, 75–81.
